# The Effects of PK11195 and Protoporphyrin IX Can Modulate Chronic Alcohol Intoxication in Rat Liver Mitochondria under the Opening of the Mitochondrial Permeability Transition Pore

**DOI:** 10.3390/cells9081774

**Published:** 2020-07-24

**Authors:** Yulia Baburina, Irina Odinokova, Olga Krestinina

**Affiliations:** Institute of Theoretical and Experimental Biophysics, Russian Academy of Sciences, 142290 Pushchino, Moscow Region, Russia; odinokova@rambler.ru (I.O.); ovkres@mail.ru (O.K.)

**Keywords:** rat liver mitochondria (RLM), chronic ethanol intoxication, translocator protein (TSPO), 2′,3′-cyclic nucleotide-3′-phosphodiesterase (CNPase), mitochondrial permeability transition pore (mPTP)

## Abstract

Decades of active research have shown that mitochondrial dysfunction, the associated oxidative stress, impaired anti-stress defense mechanisms, and the activation of the proapoptotic signaling pathways underlie pathological changes in organs and tissues. Pathologies caused by alcohol primarily affect the liver. Alcohol abuse is the cause of many liver diseases, such as steatosis, alcoholic steatohepatitis, fibrosis, cirrhosis, and, potentially, hepatocellular cancer. In this study, the effect of chronic alcohol exposure on rat liver mitochondria was investigated. We observed an ethanol-induced increase in sensitivity to calcium, changes in the level of protein kinase Akt and GSK-3β phosphorylation, an induction of the mitochondrial permeability transition pore (mPTP), and strong alterations in the expression of mPTP regulators. Moreover, we also showed an enhanced effect of PK11195 and PPIX, on the parameters of the mPTP opening in rat liver mitochondria (RLM) isolated from ethanol-treated rats compared to the RLM from control rats. We suggest that the results of this study could help elucidate the mechanisms of chronic ethanol action on the mitochondria and contribute to the development of new therapeutic strategies for treating the effects of ethanol-related diseases.

## 1. Introduction

Alcohol abuse is an important problem worldwide and is associated with various liver diseases; the mortality from these diseases is around 4% [1]. Chronic alcohol consumption leads to multiple types of liver damage and causes various alcoholic liver diseases, such as alcoholic steatohepatitis, liver fibrosis, and cirrhosis, ultimately leading to hepatocellular cancer [2,3,4]. The mitochondria are primarily affected by chronic alcohol consumption [5]. Such consumption violates the integrity and structure of the mitochondria and disrupts their normal functioning [6,7,8]. Ethanol-mediated changes include abnormalities in mitochondrial morphology. For example, enlarged, deformed mitochondria with fewer cristae can appear [9]. Chronic exposure to ethanol leads to significant disruptions in the functionality of systems of fatty acid oxidation, the urea cycle, oxidative phosphorylation, and the production of reactive oxygen species (ROS) [10,11]. It was previously shown that the rate of ATP generation significantly decreases during oxidative phosphorylation in the mitochondria of rats susceptible to chronic alcohol intoxication [12,13]. According to the results of proteomic studies, these disorders are largely associated with changes in the expression of proteins of the inner mitochondrial membrane, such as respiratory chain complexes and ATP synthase, as well as mitochondrial matrix dehydrogenases [14,15,16], and damage to them. Other mitochondrial-related processes, such as mtDNA damage, inhibition of the synthesis of mitochondrial proteins, decreases in oxidative phosphorylation, and weakening of ATP synthesis, are also involved in the pathogenesis of alcoholic diseases. [7]. The study of mitochondria under chronic alcohol consumption is important because alcohol affects the redox signaling pathways [17] and acts as a regulator of cellular Ca^2+^ homeostasis; therefore, it is involved in the mechanisms of cell death [18]. Among post-translational protein modifications, the key event is phosphorylation, including the phosphorylation of mitochondrial membrane-bound and channel-forming proteins. Thus, protein phosphorylation is the main mechanism of regulation for many signaling pathways [19,20,21]. Protein kinases can play an important role in the development of the pathogenesis of various diseases. In particular, it was shown that protein kinase B (Akt), by modulating oxidative stress, becomes involved in the processes related to alcohol damage of the heart [22]. It was also indicated that prolonged exposure to alcohol increases the phosphorylation of glycogen synthase kinase 3 (GSK-3β) in the rat nucleus accumbens [23] and mouse dorsomedial striatum [24]. In the mitochondria, the actions of these kinases are associated, in particular, with the phosphorylation of the voltage-dependent anion channel (VDAC) [25,26]. The VDAC determines the permeability of the outer mitochondrial membrane, which changes with the development of pathological conditions, including alcohol intoxication. The VDAC is closely associated with the translocator protein (TSPO), which was previously known as a peripheral benzodiazepine receptor [27]. It is assumed that TSPO is an important factor in addiction to ethanol, and modulating the conductivity of VDACs under the influence of TSPO could be a key factor in the permeability of the outer membrane. TSPO is a major component of the outer membrane of the mitochondria; it mediates various mitochondrial functions, including cholesterol transport and steroid hormone synthesis, mitochondrial respiration, apoptosis, and cell proliferation [28,29,30]. TSPO also binds the benzodiazepines associated with tolerance and addiction [31]. There are many endogenous and synthetic TSPO ligands with different chemical natures. The most commonly used high-affinity TSPO ligand from the isoquinoline family is PK11195, which has a high affinity for the receptor in the nanomolar concentration region. The same affinity for TSPO is also exhibited by high-affinity endogenous ligands, which include protoporphyrin IX (PPIX). However, recent studies have shown that ATPase may be another molecular target of PK11195 and PPIX in mitochondria [32,33]. Moreover, this link is functional, affecting both enzymatic catalysis and the regulation of mitochondrial permeability transition pore (mPTP) [32,33,34]. Therefore, it is likely that both PK11195 and PPIX have different mechanisms of action in mitochondria, and accordingly, several molecular targets for action. Both PPIX and PK11195 have the ability to modulate oxidative stress and the discovery of the mPTP [35,36,37,38]. Previously, we showed that the VDAC is co-localized with mitochondrial 2′,3′-cyclic nucleotide-3′-phosphodiesterase (CNPase) [39], which also participates in mPTP functioning [40].

To develop strategies for the treatment of alcoholism, it will be necessary to determine the molecular and cellular mechanisms underlying alcohol use disorders. In connection with this goal, in the present work, we studied the effects of PK11195 and protoporphyrin IX on the functional state of mitochondria, the parameters of mPTP opening, and the level of proteins modulating the permeability of mitochondrial membranes (TSPO, VDAC, and CNPase) in the liver mitochondria from control rats (Control RLM) and from rats chronically intoxicated with alcohol (Ethanol RLM).

## 2. Materials and Methods

### 2.1. Animals and Treatment

To study the effects of alcohol on mitochondrial functions, model experiments were performed on rats. In our experiments, we used the Lieber–DeCarly model of chronic alcohol intoxication, which allows one to achieve the consumption of alcohol at high doses [41]. Mixtures for preparing liquid nutrition were manufactured by BioServ (Frenchtown, NJ, USA). The control diet contained fats, proteins, carbohydrates, trace elements, and vitamins, with 18% of the total calories coming from proteins, 35% coming from fats, and 47% coming from carbohydrates. In the alcohol diet, 36% of the calories from the carbohydrate components were replaced by calories from ethanol, the concentration of which was 5% in the final diet. This model used isocaloric pair-feeding of the animals. For this process, male rats of the same age and weight, divided into pairs, were kept in separate cages equipped with special graduated drinking bowls, without access to water and solid food. Eight rats (aged two months) were used in the experiments, with four in each group. Rats that received an alcoholic diet had free access to food throughout the day, and the control rats received an amount of food equivalent to that consumed by their paired alcoholic rats; food consumption was measured daily. Over a 10-day period of habituation, the rats received a gradually increasing amount of ethanol (0, 1%, 2%, 3%, 4%, and 5%) in their food, and then all alcoholic rats received food containing 5% ethanol for 8 weeks. At the start of the experiment, the average weight of the rats was 167.87; during the experiment, the weight gain was 163.12 ± 11.55 g. The rats consumed an average of 60–80 calories daily, and alcoholic rats received 14.86 ± 16.25 g of ethanol per 1 kg rat weight, which is consistent with the published data.

The complete data of the body mass of each animal before and after treatment, as well as the weights of the livers and average values, are presented in Table 1.

All experiments were performed in accordance with the Regulations for Studies with Experimental Animals (Decree of the Russian Ministry of Health of 12 August 1997, No. 755). The protocol was approved by the Commission on Biological Safety and Ethics at the Institute of Theoretical and Experimental Biophysics, Russian Academy of Sciences (March 2019, protocol N18/2019).

### 2.2. Isolation of Rat Liver Mitochondria and the Outer Mitochondrial Membrane

Rat liver mitochondria (RLM) were isolated from rats by the standard method using a homogenization medium containing 210 mM mannitol, 70 mM sucrose, 1 mM ethylene glycol-bis(β-aminoethyl ether)-*N*,*N*,*N*′,*N*′-tetraacetic acid (EGTA), 0.05% bovine serum albumin fraction V, and 10 mM Tris (pH 7.3). The homogenate was centrifuged at 800× *g* for 10 min to pellet the nuclei and damaged cells. The supernatant containing the mitochondria was centrifuged for 10 min at 9000× *g*. Sedimented mitochondria were washed twice in a medium without EGTA and BSA for 10 min at 9000× *g* and resuspended in the same medium. The outer membranes of the rat liver mitochondria were obtained according to Rice and Lindsay [42]. In total, 100 mg of mitochondrial protein was suspended with 2.5 mL of KH_2_PO_4_ (20 mM) pH 7.4. The suspension was sonicated at 70–80 Wt for 15 s, followed by a 15 s pause. This procedure was repeated three times. Then, the suspension was centrifuged at 6500× *g* for 7 min. The pellet was resuspended with 2.5 mL KH2PO4 (20 mM) pH 7.4. Sonication and centrifugation were repeated. After two centrifugations, the supernatants were combined and centrifuged at 105,000× *g* for hour. The obtained pellet (outer mitochondrial membrane) was resuspended with KH_2_PO_4_ (20 mM) pH 7.4. Then, the protein concentration was determined using a Bradford assay.

### 2.3. Evaluation of Mitochondrial Functions

Mitochondria (1 mg protein/mL) were incubated at 25 °C in a medium containing 125 mM KCl, 10 mM Tris (pH 7.4), and 2 mM K_2_HPO_4_. In the experiments, glutamate (5 mM) and malate (5 mM) were used as respiratory substrates. The oxygen consumption rates (V_st.2_, V_st.3_, and V_st.4_; ng-atom O min^−1^ mg^−1^ of protein) were evaluated with the Record program (Pushchino, Russia). The change in the rate of oxygen consumption in different states, the rate of state 2 (V_st.2_), the rate of state 3 (V_st.3_), the rate of state 4 (V_st.4_), and the rate of uncoupled respiration (V_u_) were calculated as the change in the rate of oxygen consumption per minute per milligram of protein. The respiratory control index (RCI) was measured in a closed chamber after the addition of 150 μM adenosine diphosphate (ADP) to RLM and was calculated as the ratio of V_st.3_ to V_st.4_. The phosphate to oxygen (P/O) ratio was calculated as the ratio of the amount of added ADP to the amount of O_2_ needed to convert ADP to ATP.

The Ca^2+^ and TPP^+^ flows of RLM were determined with TPP^+^- and Ca^2+^-sensitive electrodes (Nico, Russia) in a 1 mL measuring chamber [35]. The mitochondria (2 mg protein/mL) were incubated at 25 °C in a medium containing 125 mM KCl, 10 mM Tris (pH 7.4), and 2 mM K_2_HPO_4_; glutamate (5 mM) and malate (5 mM) were used as substrates. The mPTP opening in RLM was induced by a threshold Ca^2+^ concentration (the first addition of Ca^2+^ contained 50 nmol Ca^2+^ per mg of protein, while the second and third contained 90 nmol Ca^2+^ per mg of protein). The parameters of Ca^2+^ transport, such as the calcium retention capacity (CRC—concentration of Ca^2+^ added to the mitochondrial suspension in which the Ca^2+^ ions accumulating in the mitochondria induce the mPTP to open) and the lag phase (the time between influx and efflux, s) were measured. The Ca^2+^-induced dissipation of the membrane potential was measured as the TPP^+^ efflux rate (V^TPP+^_out_, nmol min^−1^ mg^−1^ of protein). To study the influence of PK11195 and PPIX, they were pre-incubated with the mitochondria for 10 min at appropriate concentrations (5 µM PPIX and 100 nM PK11195).

The swelling of the RLM was measured by changes in the light scattering in the mitochondrial suspension at 540 nm (A540) and 25 °C using a Tecan I-Control infinite 200 spectrophotometer. The standard incubation medium for the swelling assay contained 125 mM KCl, 10 mM Tris (pH 7.4), 2 mM KH_2_PO_4_, and 5 mM glutamate, with 5 mM malate as a substrate. The concentration of the mitochondrial protein in each well was 0.5 mg protein/mL. Swelling was initiated via the addition of 300 nmol of Ca^2+^ per mg of protein. The swelling process was characterized by the time needed to reach the half-maximal light scattering signal (T_1/2_).

### 2.4. Sample Preparation

To prepare samples to determinate the level of regulator proteins, aliquots of mitochondria in the incubated medium from the chamber (100 μL) were taken from the chamber, placed in an Eppendorf tube, and centrifuged for 3 min at 15,000× *g*. The sediments were lysed with a lysis buffer (50 mM Tris–HCl (pH 7.4), 150 mM NaCl, 1% Triton X-100, 0.1% SDS, 1 mM EDTA, 1 mM Na_3_VO_4_, and 1 mM NaF) supplemented with proteinase/phosphatase inhibitors. Then, the obtained samples were solubilized in Laemmli buffer (Bio-Rad, Hercules, CA, USA). Samples were heated to 95 °C for 5 min. Next, 20 μg of each sample was applied to the gel and subjected to electrophoresis followed by a Western blot analysis.

### 2.5. Electrophoresis and Immunoblotting of the Mitochondrial Proteins

The samples obtained were separated under denaturing conditions in 12.5% SDS-PAGE and transferred to a nitrocellulose membrane (0.2 μm pore). Precision Plus Pre-stained Standards from Bio-Rad Laboratories (Hercules, CA, USA) were used as markers. After overnight blocking, each membrane was incubated with the appropriate primary antibody. The polyclonal TSPO antibody (1:1000), monoclonal VDAC 1, and polyclonal VDAC 3 antibody (1:1000) were obtained from Abcam (Cambridge, UK); the monoclonal VDAC antibody (1:1000) was purchased from Calbiochem (San Diego, CA, USA); the polyclonal rabbit phospho-Akt (Ser473), phospho-GSK-3β (Ser9), and Akt antibodies (dilution 1:250) were obtained from Cell Signaling (Leiden, Netherlands). The monoclonal anti-CNP antibody (anti-CNP Ab) was obtained as described [43] and used at a 1:10,000 dilution, the monoclonal GSK3β antibody was used at a 1:2000 dilution, and the Tom 20 antibody (Santa-Cruz, Dallas, TX, USA) served as a loading control and was used at a 1:2000 dilution. Immunoreactivity was detected using the appropriate secondary antibody conjugated with horseradish peroxidase (Jackson Immuno Research, West Grove, PA). Peroxidase activity was detected using Enhanced chemiluminescence (ECL) reagents (Bio-Rad, Hercules, CA, USA).

### 2.6. Statistical Analysis

For the statistical analysis, the relative levels of protein density were expressed as the mean ± SD from at least three to four independent experiments. The statistical significance of the difference between the mean values was evaluated using Student’s *t*-test. A difference was considered significant at *p* < 0.05.

## 3. Results

**Figure 1:**
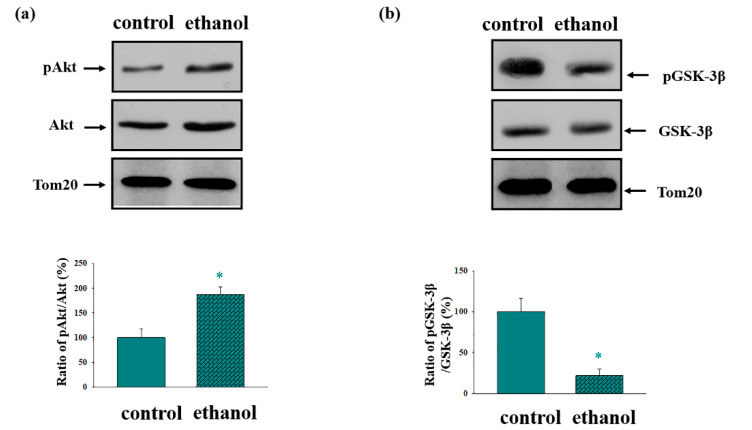
The phosphorylation states of stress-activated protein kinases (glycogen synthase kinase 3, GSK-3β, and Akt) in the rat liver mitochondria (RLM) of the control and ethanol rats. (**a**) The ratio of pAkt to total Akt. (**b**) The ratio of pGSK-3β to total GSK-3β. The upper parts represent the Western blots stained with the corresponding antibodies. The lower parts are the quantitation of immunostaining using computer-assisted densitometry. Bar graphs represent the ratios of pGSK-3β to total GSK3-3 β (**a**) and pAkt to total Akt (**b**) in the RLM of the control and ethanol (hatched columns) rats. The protein band intensity was quantified after normalization to Tom 20. The values shown are the means ± SD from three independent experiments; * *p* ≤ 0.05 compared with the control.

It is well known that protein kinases participate in different signaling pathways in the mitochondria. In particular, it was reported that the serine/threonine kinases GSK-3β and Akt are involved in the molecular pathophysiology of many mitochondria-related diseases and pathological conditions, such as Alzheimer’s disease and Parkinson’s disease, and also numerous pathologies of the heart [44,45]. Here, we examined whether chronic ethanol exposure has an effect on the amount and phosphorylation level of mitochondria-associated GSK-3β and Akt. Panel 1a illustrates an almost two-times increase in the level of Akt phosphorylation in Ethanol RLM (rat liver mitochondria isolated from rats subjected to chronic ethanol intoxication) compared to the Control RLM. At the same time, the level of phosphorylation of GSK-3β under the same conditions was five times lower (see the lower part of Figure 1b).

**Figure 2:**
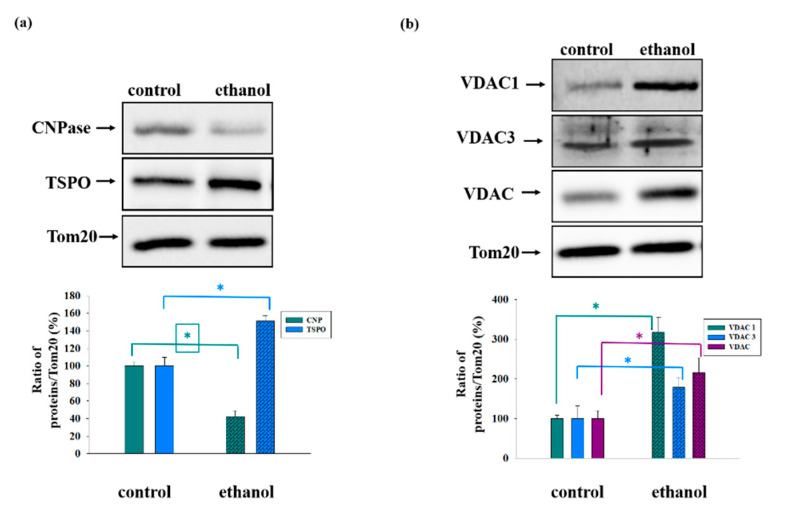
Alterations in the levels of translocator protein (TSPO), CNPase (Panel (**a**)), and voltage-dependent anion channel (VDAC 1, VDAC 3, and total VDAC) (Panel (**b**)) in the outer mitochondrial membranes of the Control and Ethanol RLM. The upper parts represent Western blots stained with the corresponding antibodies. The lower parts present the quantitation of immunostaining using computer-assisted densitometry. The protein band intensity was quantified after normalization to Tom 20. The values shown are the means ± SD from three independent experiments; * *p* ≤ 0.05 compared with the control.

We reported earlier that GSK-3β and Akt are involved in the regulation of mPTP functioning in the brain and heart mitochondria of rats [46,47]. Here, we observed changes in the levels of mitochondrial proteins that play a key role in regulating the permeability of the outer membranes in chronic alcohol intoxication. We measured the levels of TSPO, CNPase, and VDAC in the outer mitochondrial membrane of the Control and Ethanol RLM (Figure 2). As seen in Figure 2a, the level of CNPase decreased by 60% in the Ethanol RLM, while the content of TSPO increased by 50%. Panel 2b of Figure 2 reveals alterations in the level of VDAC isoforms (VDAC1 and VDAC3) and the total VDAC level. The content of all forms of VDAC in the outer mitochondrial membrane increased significantly under conditions of chronic alcohol intoxication (from 2.5 (VDAC3) to 3.5 (VDAC1) times). TSPO and the VDAC formed a strong complex [37] in the outer mitochondrial membrane. While affecting the activity of the VDAC, TSPO directly participates in modulating the permeability of the mitochondrial membranes, as well as initiating and regulating apoptosis [40,41]. Based on the above facts, we further studied the effects of the ligands of TSPO, such as endogenous PPIX and synthetic PK11195, on the functioning of the Control and Ethanol RLM.

**Figure 3:**
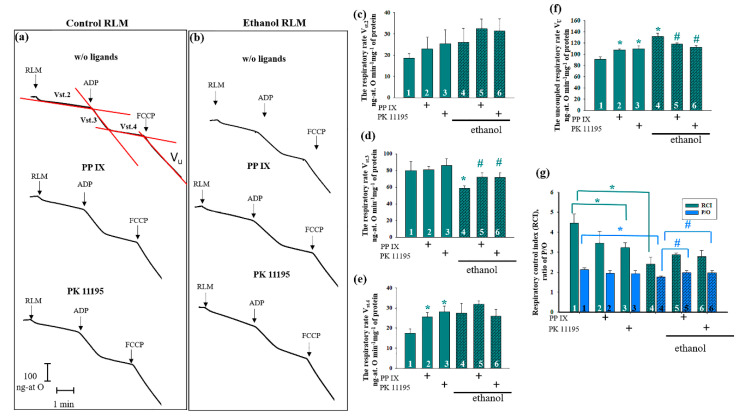
The effects of protoporphyrin IX (PPIX) and PK11195 on the respiratory activity of the Control and Ethanol RLM: curves of the respiratory activity of the Control (**a**) and Ethanol (**b**) RLM. Arrows show the times at which RLM (rat liver mitochondria), ADP (adenosine diphosphate), and FCCP (carbonyl cyanide-*4*-(trifluoromethoxy)phenylhydrazone) were added; (**c**–**f**) quantitative analysis of the RLM respiration rate in states 2, 3, and 4, as well as the uncoupled respiration rate; (**g**) respiratory control index (RCI) values and phosphate/oxygen (P/O) ratio; the shown values are the means ± SD from three independent experiments; * *p* ≤ 0.05 compared with the value in the Control RLM without additions (Column 1); # *p* ≤ 0.05 compared with the value in the Ethanol RLM without additions (Column 4).

First, we observed changes in the parameters of mitochondrial respiratory activity in the presence of PK11195 (100 nM) and PPIX (5 µM) in the Control and Ethanol RLM. Panels 3a and b show the curves of mitochondrial respiration in the control and ethanol rats, respectively. Figure 3c–f provides the oxygen consumption rates in different states. We did not observe any changes in substrate-dependent respiration (Panel C, state 2) under all experimental conditions. Further, no changes occurred in the rate of oxygen consumption (Panel D, state 3) in the presence of PK11195 and PPIX in the mitochondria of both the control and ethanol rats (Panel D, columns 2 and 3 versus 1; 5 and 6 versus 4; respectively). However, in the RLM of the ethanol rats, the rate in state 3 decreased by 25% compared to the corresponding control (Panel D, column 4 versus 1). We found an increase of approximately 20% in the state 3 oxygen consumption rates in the Ethanol RLM in the presence of PPIX and PK11195 (columns 5 and 6 versus 4). The presence of PPIX led to an increase of 30% and PK11195 led to an increase of 33% (in the respiratory rate in state 4 of the RLM of the control rats (Panel E, columns 2 and 3 versus 1). We did not observe any changes in the state 4 rates in the Ethanol RLM in the presence of both PPIX and PK11195 compared to the Ethanol RLM without additions (Panel E, columns 6 and 5 versus 4).

Figure 3f presents the uncoupled respiration rate (V_u_), a parameter reflecting the magnitude of maximum respiration. The increase of Vu in the Ethanol RLM compared to the control was 20% (Panel D, column 4 versus 1). In the Control RLM, PPIX and PK11195 caused an increase of V_u_, whereas in the Ethanol RLM, this parameter was lower by 12% in the presence of PPIX (Panel F column 5 versus 4) and by 15% in the presence of PK11195 (Panel D column 6 versus 4).

Moreover, we calculated the respiratory control index (RCI), which indicates the effectiveness of mitochondria in promoting oxidative phosphorylation and coupling between oxygen consumption and ATP production. The results are presented in Figure 3g (cyan columns). In the RLM of the control rats, the addition of PPIX caused no changes in the RCI (cyan column 2 versus 1), but in the presence of PK11195, the RCI increased by 20% (cyan column 3 versus 1). The RCI of the Ethanol RLM was two times lower than that of the control animals, but at the same time, PK11195 and PPIX had no significant influence on the RCI in the Ethanol RLM (cyan columns 6 and 5 versus 4). Figure 3f (blue columns) presents data on the efficiency of oxidative phosphorylation in the mitochondria, which is defined as the ratio of ATP to absorbed oxygen (P/O). As seen in the figure, there were no significant changes in this parameter in the presence of both PPIX and PK11195 in the Control RLM (blue columns 2, 3 versus 1), but we observed a slight decrease in the P/O ratio in the Ethanol RLM compared to the Control RLM (blue column 4 versus 1). The addition of PPIX and PK11195 to the RLM of the ethanol rats caused an increase in the P/O ratio by 18% (blue columns 5 and 6 versus 4).

**Figure 4:**
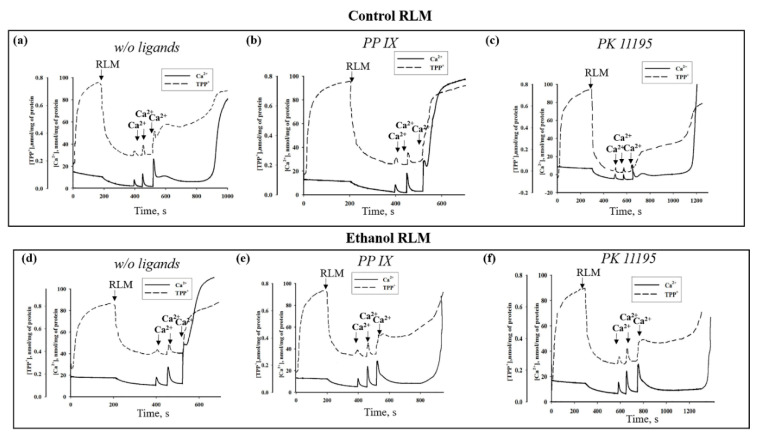
The effect of PPIX and PK11195 on the Ca^2+^ transport and membrane potential changes in the Control (**a**–**c**) and Ethanol (**d**–**f**) RLM. The arrows indicate where CaCl_2_ was added to the mitochondrial suspension. RLM were incubated in a standard medium, as described in the Materials and Methods.

Next, we examined the parameters of mPTP functioning, such as Ca^2+^ transport, membrane potential (Δψm), and calcium retention capacity (CRC) and compared them under different experimental conditions (in the RLM of the control and ethanol rats and in the presence of PPIX and PK11195). Figure 4 shows the curves of changes in the Ca^2+^ flow and Δψm. Pulses of Ca^2+^ were added to the mitochondria to reach a threshold Ca^2+^ concentration for the mPTP to open. The first two additions of Ca^2+^ (50 and 90 nmol per mg of protein) led in all cases to a stable accumulation of Ca^2+^ in the mitochondria followed by the recovery of Δψm. In the RLM of the control rats (Figure 4a), the third addition of Ca^2+^ (90 nmol per mg of protein) led to an activation of the mPTP, even after a prolonged lag phase. As seen in Figure 4, the addition of PPIX resulted in the stimulation of the mPTP to open in the Control but not in the Ethanol RLM (Figure 4b versus 4a and 4e versus 4d, respectively). We observed an acceleration of the mPTP’s opening in the Ethanol RLM compared to the Control RLM (Figure 4d versus 4a) and a deceleration of the mPTP’s opening in the presence of PK11195 in both the Control and Ethanol RLM (Figure 4c versus 4a and 4f versus 4d).

**Figure 5:**
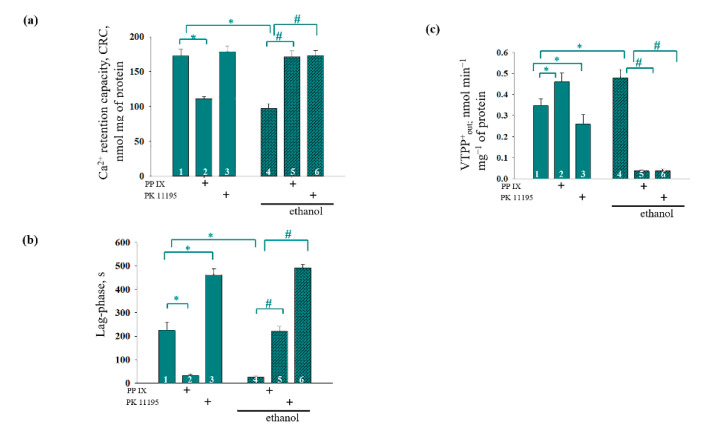
Quantitative analysis of the action of PPIX and PK11195 on the Ca^2+^-induced mitochondrial permeability transition pore (mPTP) opening in the Control and Ethanol RLM; (**a**) quantitative analysis of the CRC (calcium retention capacity) and (**b**) quantitative analysis of the lag phase; (**c**) quantitative analysis of V^TPP+^_out_. The values shown are the mean ±SD from three independent experiments; * *p* ≤ 0.05 compared with the control value in the RLM without additions (column 1); # *p* ≤ 0.05 compared with the control value in the Ethanol RLM without additions (hatched column 4).

The quantitative characteristics of these phenomena are presented in Figure 5. Figure 5a presents the quantitative changes of the CRC in the Ca^2+^-loaded mitochondria. In the RLM from the ethanol rats, the CRC was diminished by 44% compared to the Control RLM (hatched column 4 versus column 1). The addition of PPIX increased the CRC by 40% in the Ethanol RLM (hatched column 5 versus hatched column 4) and decreased it by approximately the same amount in the Control RLM (cyan column 2 versus column 1). The presence of PK11195 had no effect on the CRC in the RLM of the control rats (cyan columns 3 versus 1) but enhanced it in the Ethanol RLM (hatched columns 6 versus 4). The results showing changes in the lag phase (the time between the influx and efflux of Ca^2+^ during the last Ca^2+^ addition) generally correlate with the changes in the CRC, thereby confirming the influence of PPIX and PK11195 on the parameters of the Ca^2+^ flow in both the Control and Ethanol RLM (Figure 5b). Thus, in the RLM from ethanol rats, the lag phase was dramatically (10 times) reduced compared to the control (hatched column 4 versus cyan column 1). PPIX caused a 10-fold decrease in the lag phase in the RLM from the control rats (cyan column 2 versus 1) and an increase of the lag phase in the Ethanol RLM (2 times, hatched column 5 versus hatched column 4). The addition of PK11195 significantly increased the lag phase in both the Control and Ethanol RLM (cyan column 3 versus 1 and hatched columns 6 versus 4, respectively).

In addition, we measured the Ca^2+^-induced dissipation of membrane potential calculated as the TPP^+^ efflux rate (V^TPP+^_out_, nmol min^−1^ mg^−1^ of protein). As seen in Figure 5c, the rate of TPP^+^ efflux in the RLM from the ethanol rats increased by 30% compared to the control (hatched column 4 versus column 1). PPIX accelerated V^TPP+^_out_ by 30% in the RLM from the control rats (cyan column 2 versus black column 1), while V^TPP+^_out_ slowed by 7 times in the Ethanol RLM (hatched column 6 versus hatched column 4). PK11195 reduced V^TPP+^_out_ in the Control RLM (cyan columns 3 versus 1) and Ethanol RLM (hatched columns 6 versus 4), but in the Ethanol RLM, its effect was more pronounced (25% in the Control RLM and 7 times in the Ethanol RLM).

**Figure 6:**
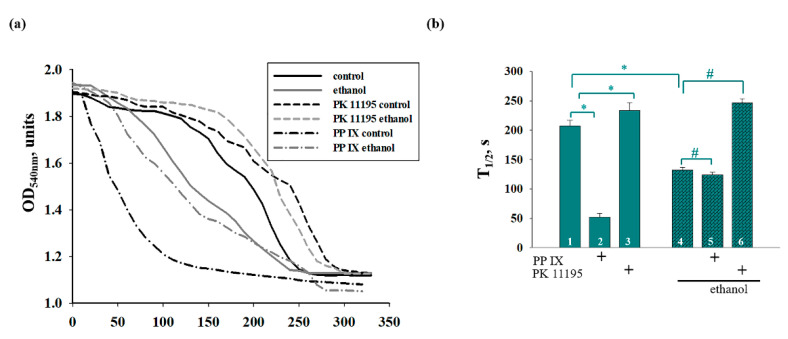
The effect of PPIX and PK11195 on the Ca^2+^-induced swelling of the Control and Ethanol RLM. (**a**) Curves of RLM swelling. (**b**) Average half-times (T_1/2_) of swelling. The values shown are the mean ±SD from three independent experiments; * *p* ≤ 0.05 compared with the control value in the Control RLM without additions (cyan column 1), # *p* ≤ 0.05 compared with the control value in the Ethanol RLM without additions (hatched column 4).

The addition of Ca^2+^ at a threshold concentration to the mitochondrial suspension incubated in the standard medium caused a decrease in light scattering, indicating a Ca^2+^-dependent swelling of the mitochondria. Next, we compared the swelling of the Control and Ethanol RLM in the presence of PPIX and PK11195. Figure 6a shows the standard curves of RLM swelling under different experimental conditions. The amount of added Ca^2+^ needed to induce swelling was 230 nmol per mg of protein in each case, which corresponds to the control amount of Ca^2+^ shown in Figure 4. Figure 6b presents the average half-time of Ca^2+^-activated mitochondrial swelling (the time needed to reach the half-maximal light scattering signal, T_1/2_). In the Ethanol RLM, T_1/2_ was reduced by 40% (hatched column 4 versus cyan column 1), indicating a greater sensitivity to Ca^2+^ overloading and mPTP initiation in the Ethanol RLM. PPIX reduced the T_1/2_ in the Control RLM by 4 times (cyan column 2 versus cyan column 1) but did not change T_1/2_ in the Ethanol RLM (hatched columns 5 versus 4) compared to the corresponding control. In the presence of PK11195, T_1/2_ was slightly increased in the Control RLM (by 15%, cyan columns 3 versus 1) and dramatically increased in the Ethanol RLM (hatched columns 6 versus 4) compared to the corresponding control.

**Figure 7:**
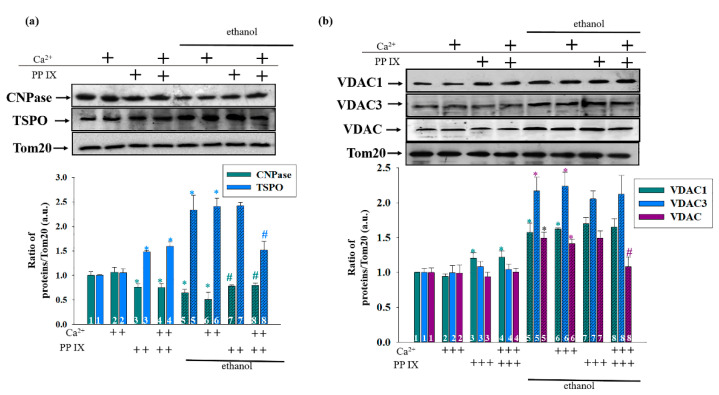
The effect of PPIX on the level of mitochondrial proteins. The upper parts represent Western blots stained with the corresponding antibodies. The lower parts represent the quantitation of immunostaining using computer-assisted densitometry. (**a**) The alterations in CNPase and TSPO levels; (**b**) the alterations in VDAC 1, VDAC 3, and total VDAC levels. The protein band intensity was quantified after normalization to Tom 20. The values shown are the means ±SD from three independent experiments. The level of each protein in the Control RLM without additions was taken as 1. * *p* ≤ 0.05 compared with the corresponding value in the Control RLM without PPIX additions with the opened and closed mPTP, respectively (columns 3 and 5 vs. column 1 for the mPTP closed state and columns 4 and 6 vs. 2 for the mPTP opened state), # *p* ≤ 0.05 compared with the corresponding value in the Ethanol RLM without PPIX additions (hatched columns 7 vs. 5 in the closed mPTP and 8 vs. 6 in the opened mPTP).

Considering the possible ability of the key proteins of the outer membrane to regulate the function of the mPTP, we investigated changes in the levels of these proteins in the Control and Ethanol RLM under conditions of the open/closed mPTP in the presence of PPIX and PK11195. Figure 7 shows alterations in the content of CNPase, TSPO (Figure 7a), and VDAC isoforms (Figure 7b) under the action of PPIX. We compared data on PPIX’s effect on the protein levels in both the closed mPTP (columns 3 versus 1 for the Control RLM; and columns 7 versus 5 for the Ethanol RLM) and opened mPTP (columns 4 versus 2 for the Control RLM and 8 versus 6 for the Ethanol RLM). Separately, we observed the changes in protein content in the Ethanol RLM compared to the Control RLM (columns 5 versus 1 for the opened mPTP and 6 versus 2 for the closed mPTP). Figure 7a (the lower part) presents a diagram showing the quantitative characteristics of the changes in the levels of TSPO and CNPase. As shown above, the level of CNPase in the Ethanol RLM was lower than that in the Control RLM by approximately 40% in both the closed (hatched cyan column 5 versus cyan column 1) and opened mPTP (hatched cyan column 6 versus cyan column 2). The addition of PPIX, when the mPTP was closed, caused a reduction in CNPase content by 20% in the RLM from the control rats (cyan columns 3 versus 1) and a slight increase in the Ethanol RLM (hatched cyan columns 7 versus 5). For the opened mPTP, the presence of PPIX decreased the level of CNPase by 28% in the Control RLM (cyan columns 4 versus 2) and increased it by 30% in the Ethanol RLM (hatched cyan columns 8 versus 6). The blue columns in Figure 7a represent the changes in the TSPO levels in our experimental conditions. In the RLM from the ethanol rats, the level of TSPO was more than double that of the control in both the closed (hatched blue column 5 versus blue column 1) and opened mPTP (hatched blue column 6 versus blue column 2). Under the action of PPIX, the level of TSPO in the Control RLM increased by approximately 50% when the mPTP was closed (blue columns 3 versus 1) or opened (blue columns 4 versus 2). In the Ethanol RLM, PPIX caused no changes when the mPTP was closed (hatched blue columns 7 versus 5) and showed a decrease of 40% when the mPTP was opened (hatched blue columns 8 versus 6).

The alterations in the level of different isoforms of VDAC are shown in Figure 7b. In the Ethanol RLM, the level of VDAC1 (cyan columns) was increased by 50% compared to the control in both the closed (hatched cyan column 5 versus cyan column 1) and opened mPTP (hatched cyan column 6 versus cyan column 2). Under the action of PPIX, the level of VDAC1 was enlarged in the Control RLM by 18–20% when the mPTP was closed (cyan columns 3 versus 1) and opened (cyan columns 4 versus 2). In the Ethanol RLM, PPIX had no effects in both the closed and opened mPTP (hatched cyan columns 7 versus 5 and 8 versus 6, respectively). The blue columns in Figure 7b indicate changes in the level of VDAC3. We observed changes only in the Ethanol RLM compared to the control RLM without PPIX addition (a 2.3 times increase in the VDAC3 level, hatched blue column 5 versus blue 1 and hatched blue 6 versus blue 2) in both the opened and closed mPTP. The presence of PPIX did not cause any changes in the VDAC3 levels in both the Control and Ethanol RLM. The content of total VDAC (violet columns) increased by 50% in the Ethanol RLM compared to the Control RLM when the mPTP was both opened and closed (hatched violet column 5 versus violet column 1 and hatched violet column 6 versus violet column 2). PPIX did not affect the RLM from the control (violet columns 3 versus 1 and 4 versus 2) and ethanol rats when the mPTP was closed (hatched violet columns 7 versus 5). However, when the mPTP was opened, the level of total VDACs decreased in the RLM from the ethanol rats by 28% (hatched violet columns 8 versus 6) relative to the corresponding control.

**Figure 8:**
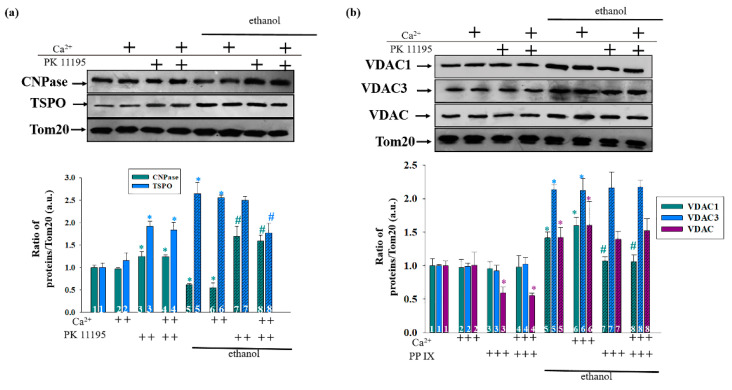
The effect of PK11195 on the level of mitochondrial proteins, which are regulators of the mPTP. The upper parts represent Western blots stained with the corresponding antibodies. The lower parts represent quantitation of immunostaining using computer-assisted densitometry. (**a**) Alterations in CNPase and TSPO levels; (**b**) alterations in VDAC 1, VDAC 3, and total VDAC levels. The protein band intensity was quantified after normalization to Tom 20. The values shown are the mean ± SD from three independent experiments. The levels of each protein in the Control RLM without additions were taken as 1. * *p* ≤ 0.05 compared with the corresponding value in the Control RLM without PK11195 additions for the opened and closed mPTP, respectively (columns 3 and 5 vs. column 1 for the closed state mPTP and columns 4 and 6 vs. 2 for the mPTP with an opened state), # *p* ≤ 0.05 compared with the corresponding value in the Ethanol RLM without PK11195 additions (hatched columns 7 vs. 5 for closing mPTP and 8 vs. 6 for opening mPTP).

Figure 8a presents data on the alterations in the content of CNPase under the action of PK11195. The changes in the Ethanol RLM compared to the Control RLM (columns 5 versus 1 and 6 versus 2) in this figure coincide with the results described previously (Figure 7a); therefore, to avoid repetition, we will focus here on the action of PK11195. In the RLM from the control rats, the level of CNPase in the presence of PK11195 increased by 20% when the mPTP was both closed (cyan columns 3 versus 1) and opened (cyan columns 4 versus 2). Conversely, in the RLM from the ethanol rats, the action of PK11195 caused a threefold increase in the content of CNPase in both states of the mPTP (hatched cyan columns 7 versus 5 and 8 versus 6, respectively).

In the Control RLM, the effect of PK11195 on the content of TSPO was almost doubled when the mPTP was both opened and closed (blue columns 3 versus 1 and 4 versus 2, respectively). In the Ethanol RLM, we did not observe any changes in the presence of PK11195 when the mPTP was closed (hatched blue columns 7 versus 5); however, it decreased by 30% when the mPTP was opened (hatched blue columns 8 versus 6).

The effect of PK11195 on the levels of different VDAC isoforms is shown in Figure 8b. The differences in the levels of VDAC isoforms in the RLM from the control and the ethanol rats without PK11195 are similar to the results in Figure 7b. In the presence of PK11195, the level of VDAC1 in the Control RLM did not change when the mPTP was both closed (cyan columns 3 versus 1) and opened (cyan columns 4 versus 2). In the Ethanol RLM, the addition of PK11195 decreased the level of VDAC1 by 48–50% in both the closed and opened mPTP (hatched cyan columns 7 versus 5 and 8 versus 6, respectively). Changes in the level of VDAC3 (blue columns) are shown in Figure 8b. The presence of PK11195 did not change the level of VDAC3 in both the Control and Ethanol RLM. Finally, the level of total VDACs (dark gray columns) decreased by almost two times in the RLM from the control rats when the mPTP was both closed and opened (violet columns 3 versus 1 and 4 versus 2). However, the level of total VDACs was not altered under the action of PK11195 in the RLM from the ethanol rats when the mPTP was closed (hatched violet columns 8 versus 6 and 7 versus 5).

## 4. Discussion

Although chronic alcoholism causes numerous lesions to various organs and cells of the body, resulting in a variety of diseases, the main metabolism of ethanol occurs in the liver. This is because the liver is the first barrier in the pathway of toxic substances. The formation of a highly reactive intermediate product, acetaldehyde, also occurs in the liver. This product initiates the free radical processes, which leads to damage of the hepatocyte cell membranes [48]. The main cause of pathological changes in chronic alcoholism is mitochondrial dysfunction, particularly impairment of the pro-apoptotic signaling pathways, oxidative stress, and as a result, the disturbance of anti-stress protective mechanisms [7]. Different mitochondrial functions are modulated by the phosphorylation/dephosphorylation of mitochondrial membrane-bound and channel-forming proteins [19,20,21,35]. Studies in other laboratories have shown that many key mitochondrial proteins are phosphorylated by GSK-3β [49,50,51] and Akt [49,52]. Akt, a serine/threonine protein kinase, is a key regulator of cell proliferation and survival. Akt is rapidly activated in response to oxidative stress and thus phosphorylates GSK-3β at Ser9 [53]. Here, we demonstrated an increase in the level of Akt phosphorylation in the Ethanol RLM compared to the Control RLM, and in turn, a decrease in GSK-3β phosphorylation. It should be noted that Akt phosphorylates GSK-3β at Ser9 [51], causing its inactivation, so we expected an increase in the level of GSK-3β as well. Likely, the level of GSK-3β phosphorylation is regulated by the action of other protein kinases. There is evidence indicating the regulation of GSK-3β by protein kinase A, as well as ribosomal s6 kinase [54,55]. Thus, since the phosphorylation of Akt leads to Akt activation [56], and conversely, a reduction in GSK-3β phosphorylation at Ser9 leads to an activation of GSK-3β [57], we suggest that ethanol-induced intoxication results in the activation of GSK-3β in RLM.

The induction of mPTP leads to an increase in the permeability of the inner mitochondrial membrane, as well its depolarization, swelling, and damage to the outer membrane. There is also evidence of the impaired functioning of the mPTP, which is one of the most important characteristics of the mitochondria, in chronic alcoholism [58,59]. Studies by King et al. showed that chronic ethanol consumption initiates the formation of the mPTP, which leads to mitochondrial dysfunction and cell death [60]. However, the mechanisms responsible for this effect remain poorly defined.

It should be noted that despite intensive studies, the composition of the mPTP has not yet been determined. Among the mPTP’s most important regulators is the VDAC located on the outer mitochondrial membrane [35,61]. The VDAC forms a durable complex with TSPO on the outer membrane [62] and co-precipitates with CNPase [39], which can also regulate the mPTP’s functions [40]. The TSPO was previously considered to be a regulator of the mPTP; however, recent studies indicated that the TSPO does not directly affect the parameters of the mPTP. In particular, it was shown that the regulation of the mPTP by the outer membrane does not involve the TSPO [63]. At the same time, there is evidence indicating the direct involvement of the TSPO in the regulation of the mPTP in various pathologies, in particular, ischemia/reperfusion in cardiomyocytes [64,65], as well as in the lung cancer cells in cases of damage caused by cigarette smoke [66]. Our recent studies have shown that mitochondria isolated from TSPO knockdown glioma cells were unable to accumulate and retain the same amounts of Ca^2+^ (before mPTP opening) as mitochondria isolated from wild-type glioma C6. This indicates that the suppression of TSPO expression is related to decreased Ca^2+^ capacity and a reduction of threshold calcium concentration in knockdown mitochondria, which leads to the induction of mPTP opening (unpublished data). Thus, we and other researchers [64,67] assume that the TSPO may have different functions in mitochondria in normal cells and in various pathologies. This assumption also refers to its effect on the mPTP.

We found the opposite effect of ethanol on the level of CNPase and TSPO in the outer membrane, since the CNPase level decreased. The TSPO level, on the other hand, increased compared to the control value. Earlier, we noted a decrease in the CNPase level in rat brain mitochondria with aging [68], and conversely, an increase with acute heart failure [69]. This is apparently due to the different mechanisms underlying the development of acute and chronic lesions in the mitochondria and different ways that CNPase participates in these processes. For the correlation between the levels of expression of CNPase and TSPO, we previously observed a similar effect in mitochondria isolated from Glioma C6 cells (not published), which possibly indicates that CNPase is involved in the protein synthesis in the mitochondria and regulates the levels of the mitochondrial pool in the TSPO. Considering the RNA-binding properties of CNPase and its participation in protein synthesis, we suggest a novel functional interaction of the TSPO and CNPase in the mitochondria, which might be related to the modulation of nuclear gene expression. Moreover, we observed an increase in the level of different isoforms of VDACs (VDAC1, VDAC3, and total VDAC) on the outer membrane of Ethanol RLM (Figure 2b). The greatest (more than threefold) increase was found in the level of VDAC1. VDAC1 is the most widely distributed multi-functional protein, which is expressed in the mitochondria and other cell compartments. This protein regulates the main metabolic and energetic functions of the cell, including Ca^2+^ homeostasis, oxidative stress, and mitochondria-mediated apoptosis [70]. The VDAC3 level was also increased in Ethanol RLM, as was the total VDAC level (recognized all three isoforms of the VDAC). In recent years, studies concerning the role of VDAC3 in various mitochondria-mediated pathways have been published. Reina et al. reported that VDAC3 is a potential marker of the mitochondrial status in cancer and other pathologies [71], and it is also an indicator of the oxidative status in the mitochondria [72].

Taking into account the present data on changes in the levels of TSPO, VDAC and CNPase under the action of ethanol in the outer mitochondrial membrane, as well as our previous data concerning mPTP regulation by PK11195 and PPIX [35,73], in this work, we tested the influence of these two substances on mPTP parameters and the expression of mPTP regulators on the Control and Ethanol RLM. We found that both PPIX and PK11195 can enhance the mitochondrial phosphorylation-coupled respiration rate inhibited by ethanol (Figure 3c, V_st.3_). A partial uncoupling of mitochondrial respiration (Figure 3d) induced by PPIX and PK11195 was revealed in the Control RLM but not in the Ethanol RLM. Apparently, a special mechanism of action operates in the mitochondria of rats exposed to the chronic effect of ethanol that is different from the mechanism of action under control conditions. This assumption was confirmed by the data on the mPTP parameter changes in the Ethanol RLM compared to the Control RLM. PPIX reduced the CRC and lag phase prior to the release of Ca^2+^ from the mitochondria, the acceleration of mitochondrial swelling, and the drop in membrane potential (i.e., it stimulated the mPTP to open in the RLM of the control rats). In the RLM from the ethanol rats, the PPIX effect was exactly the opposite—i.e., it inhibited the opening of the mPTP. PK11195 significantly enhanced the inhibitory effect on the mPTP opening in the Ethanol RLM compared to the corresponding control. Thus, in the RLM from the control rats, PK11195 caused a twofold increase in the lag phase, but in the RLM from the ethanol group, this increase was 10-fold. The PK11195-induced acceleration of the membrane potential dissipation in the Ethanol RLM was 5 times stronger than that in the control ones, and the inhibition of the mitochondrial swelling rate increased by 1.7 times. It should be noted that the Bernardi group showed that the effects of PK11195 and PPIX on the functioning of the mPTP are not TSPO-dependent [63]. There is an assumption that there are both TSPO-dependent and TSPO-independent ligand effects, and in some pathologies TSPO-independent effects predominate. Since we showed a change (increase) in the effects of PK11195 and PPIX in ethanol mitochondria compared with the control RLM, we suppose an existence of a special mechanism in chronic alcohol intoxication that activates the TSPO-dependent pathways of ligand action. Moreover, the effect of ligands may also depend on their concentration. Thus, we previously showed that different concentrations of PK11195 can have opposite effects on the mPTP [35,74], which is apparently associated with different mechanisms of action. The ligands in the nanomolar concentration range studied in this work are likely to have a greater specificity for the TSPO, while higher concentrations can lead to other, TSPO-independent effects of both PPIX and PK11195. Thus, the work of Glik et al. shows the possibility of the action of PPIX and PK11195 on the operation of the mPTP via the F1F0-ATPase, which is currently considered to be a structural regulator of the pore [33,75].

We suggest that ethanol induces changes in the expression of proteins regulating the mPTP’s functions, which leads to impairment in the efficiency of the mitochondria. We observed the opposite effect of PPIX on changes in the level of TSPO and CNPase in the RLM from both the control and the ethanol rats under mPTP opening conditions (Figure 7a). In turn, the effect of PK11195 on the change of TSPO and CNPase level was stronger in the RLM from the ethanol rats when the mPTP was opened or closed. These data are in good agreement with the data obtained from measurements of the most important parameters of the mitochondria (Figure 3, Figure 4, Figure 5 and Figure 6).

In addition, we found an inverse relationship between the levels of these proteins themselves (an increase in the content of TSPO was accompanied by a decrease in the CNPase level and vice versa). Moreover, we showed an increase in the level of VDAC1, VDAC3, and total VDAC in the Ethanol RLM compared to the control when the mPTP was closed and opened. However, almost no changes caused by the addition of PPIX and PK11195 to the Ethanol RLM were observed, except for PK11195 abolishing the ethanol-dependent increase in the level of VDAC1. Presumably, they either indirectly influence the levels of the VDAC channels or do not affect them at all.

Earlier, we have showed a decrease in the CNPase level in the mitochondria during aging [68,76] and suggested that CNPase is able to protect the mitochondria from damage, possibly regulating the level of 2′,3′-cAMP. A decrease in the CNPase level (as a result of age-related changes or under the influence of ethanol) apparently leads to a loss of resistance of the mitochondria to stress and damage. A negative correlation between the CNPase and TSPO levels in HL-60 [77] and glioma C6 cells (unpublished data) was observed. Here, a similar inverse correlation was observed between the changes of the TSPO and CNPase levels. There is significant evidence of an increase in TSPO expression in various pathologies, such as in brain tumors [78,79,80,81], brain damage [81,82,83,84], and cancer cell proliferation [85,86,87,88]. However, information on the role of the TSPO in the pathogenesis of mitochondrial diseases remains extremely scarce. Taken together, these findings suggest there is a functional interaction between the TSPO and CNPase in the mitochondria in response to degenerative ethanol-induced changes that is possibly regulated by protein kinases. Additional studies are clearly required to clarify the mechanism of this interaction and its functional significance. However, our research suggests that the TSPO and CNPase are potential targets for therapeutic regulations. PPIX and PK11195 are able to influence the function of this system and could thus be pharmacological agents for the targeted treatment of alcohol intoxication disorders.

## 5. Conclusions

In conclusion, chronic ethanol intoxication in the liver mitochondria causes an increase in sensitivity to Ca^2+^, changes in the level of Akt and GSK-3β phosphorylation, facilitation of mPTP induction, and strong alterations in the expression of mPTP regulators (namely, a decrease in CNPase levels and an increase in the levels of the TSPO and VDAC). We also demonstrated the influence of PK11195 and PPIX on these processes and found an increase in the efficiency of their action in the Ethanol RLM compared to the Control RLM. Taking into account all the data, we suggest the existence of a compensatory system for the functioning of the TSPO and CNPase in mitochondria, where a decrease in the CNPase level leads to the activation of the mPTP and an increase in TSPO content. The data obtained suggest that the both PPIX and PK11195 are involved in the regulation of this system. The mechanism of its function remains unclear and requires further studies; however, it is now possible to suggest the application of PPIX and PK11195 for the treatment of diseases associated with chronic alcohol intoxication.

## Figures and Tables

**Table 1:** Changes in weight characteristics after the ethanol treatment of rats.

	Control	Ethanol	Control	Ethanol	Control	Ethanol
Pairs No	Body weight before treatment, g	Body weight before treatment, g	Body weight after treatment, g	Body weight after treatment, g	Liver weight, g	Liver weight, g
Pair 1	165.0	165.0	346.0	316.0	10.3	11.7
Pair 2	173.0	178.0	334.0	322.0	10.6	11.4
Pair 3	169.0	170.0	338.0	326.0	10.9	11.8
Pair 4	163.0	160.0	343.0	306.0	10.4	11.4
mean	167.5 ± 4.4	168.2 ± 7.7	340.2 ± 5.3	317.5 ± 8.7	10.5 ± 0.3	11.6 ± 0.2
